# Beyond adrenal suppression: a comprehensive post-marketing safety profile of etomidate from real world data

**DOI:** 10.3389/fmed.2026.1780171

**Published:** 2026-07-07

**Authors:** Huiyi Deng, Wujiang Lai, Ying Xiang, Zewen Wang, Jiemei Liang, Yiwen Zhang, Jiale Liang

**Affiliations:** 1 Department of Gynecology, the Eighth Affiliated Hospital of Southern Medical University (The First People's Hospital of Shunde), Foshan, Guangdong, China; 2 Senior Department of Traditional Chinese Medicine, the Sixth Medical Center of PLA General Hospital, Beijing, China; 3 Department of Anesthesiology, the Eighth Affiliated Hospital of Southern Medical University (The First People's Hospital of Shunde), Foshan, Guangdong, China

**Keywords:** adverse events reports, anesthesia, drug safety profile, etomidate, pharmacovigilance research

## Abstract

**Background:**

Etomidate is a widely used intravenous anesthetic, yet concerns persist regarding its potential for adverse drug reactions (ADRs) beyond well-known adrenal suppression. Pre-marketing trials may not fully capture its long-term or rare safety profile, necessitating continuous post-marketing surveillance.

**Methods:**

A retrospective pharmacovigilance study was performed based on the U.S. FDA Adverse Event Reporting System (FAERS) database (Q1 2004–Q2 2025). Disproportionality analysis using ROR, PRR, BCPNN and EBGM algorithms was conducted and the corresponding 95% CIs were calculated. ADRs meeting the criteria of all four algorithms were considered positive signals significantly associated with the target drug.

**Results:**

Totally 318 ADRs were acquired, of which 59 preferred terms (PTs) and two system organ classes (SOCs) met four algorithm criteria. In addition to known cardiovascular, muscular and adrenal related PTs, novel positive signals included cardiac arrest (*n* = 33), seizure (*n* = 12), drug abuse (*n* = 10), hypokalaemia (*n* = 9) and bruxism (*n* = 9) were found to be correlated with etomidate. As for SOCs, cardiac disorders (*n* = 100) remains significantly associated with etomidate while endocrine disorders (*n* = 38) was uncovered as a novel signal.

**Conclusions:**

This study confirmed known risks and identified novel ADRs of etomidate. These findings underscore the need for heightened clinical vigilance and further investigation into its long-term safety profile.

## 1 Background

Etomidate, an intravenous anesthetic agent, is widely used for the induction of anesthesia due to its rapid onset and minimal cardiovascular effects, which makes it particularly useful in high-risk patients and critical care settings (1–3). It shows several pharmacological advantages over other anesthetics, such as reduced hemodynamic instability and a short duration of action (4–7). However, despite its benefits, recent evidence has highlighted significant concerns regarding the adverse effects of etomidate, particularly its impact on adrenal function and other potential complications (8–10). Besides, in some countries like China, there's an issue with the misuse of etomidate, which can be fatal (11, 12). It was reported that etomidate has been added to e-cigarettes (13). Abusing etomidate can lead to addiction and severely affect individual health, causing nausea, vomiting, dizziness, blurred vision, slurred speech, hand tremors, fatigue, irritability, memory loss, and even death (14). China has labeled etomidate as a class II psychoactive substance with drug like properties.

The primary concern with etomidate was its ability to inhibit 11β-hydroxylase, an enzyme crucial for adrenal cortisol synthesis (15). This inhibition can lead to a transient but clinically relevant suppression of adrenal cortisol production, which may compromise the stress response in critically ill patients who are already vulnerable to adrenal insufficiency (16, 17). This effect has been associated with increased morbidity and prolonged recovery times (9, 18, 19), particularly in patients undergoing major surgeries or those with pre-existing adrenal conditions. In addition to adrenal suppression, etomidate has been reported to be associated with other adverse effects such as injection site reactions, myoclonus and hypersensitivity reactions in the post-marketing surveillance (4). While these effects are generally considered to be mild, they can impact patient comfort and recovery, and may complicate the management of anesthesia in sensitive populations. Continuous update of the drug safety profile is necessary for better regulation of the drug.

The FDA Adverse Event Reporting System (FAERS) is a comprehensive database managed by the U.S. Food and Drug Administration (FDA) that collected and analyzed reports of adverse events and medication errors related to drugs and biologics (20). FAERS is a vital resource for monitoring the safety of pharmaceutical products and ensuring public health. It contains data from healthcare professionals, patients, and manufacturers, providing insights into potential safety issues and enabling the FDA to take regulatory actions if necessary. The database includes information on patient demographics, the nature of the adverse events, and the drugs involved, facilitating ongoing safety surveillance and research (21–23).

This paper aims to provide a comprehensive review of the adverse effects associated with etomidate based on the data from FAERS database, with a focus on its impact on adrenal function and other potential complications. By performing the real-world disproportionality analysis, we seek to offer a clearer understanding of the novel potential risks associated with etomidate and to propose strategies for mitigating these risks in clinical practice.

## 2 Methods and materials

### 2.1 . Data sources

#### 2.1.1 Acquisition of the data about drug AEs

Between the first quarter of 2004 and the second quarter of 2025, AEs associated with etomidate, reported by healthcare professionals, pharmaceutical companies, patients, and others, were obtained from the FAERS database. This database has been recognized as a global reporting system known for its extensive data and standardized approach. The information was updated quarterly and publicly accessible. Our study was performed based on the publicly available database and it was not the clinical trial. Therefore the clinical trial number was not applicable. Reported AEs in the FAERS database were coded using PTs from the Medical Dictionary for Regulatory Activities (MedDRA), which were logically organized into five hierarchical levels. PTs represented specific medical concepts, including signs, symptoms, and disease diagnoses. The FAERS database files, updated quarterly since 2004, were published on the FDA's website (https://open.fda.gov/data/faers). These files consisted of seven data sheets covering various aspects such as data sources (reporting region, reporter's occupation, and report year), patient characteristics (age and gender), drug administration details (indications, dosage, and route), the date and outcome of AEs, and more.

#### 2.1.2 Standardization of the terms of drug names and AEs

**Figure 1:**
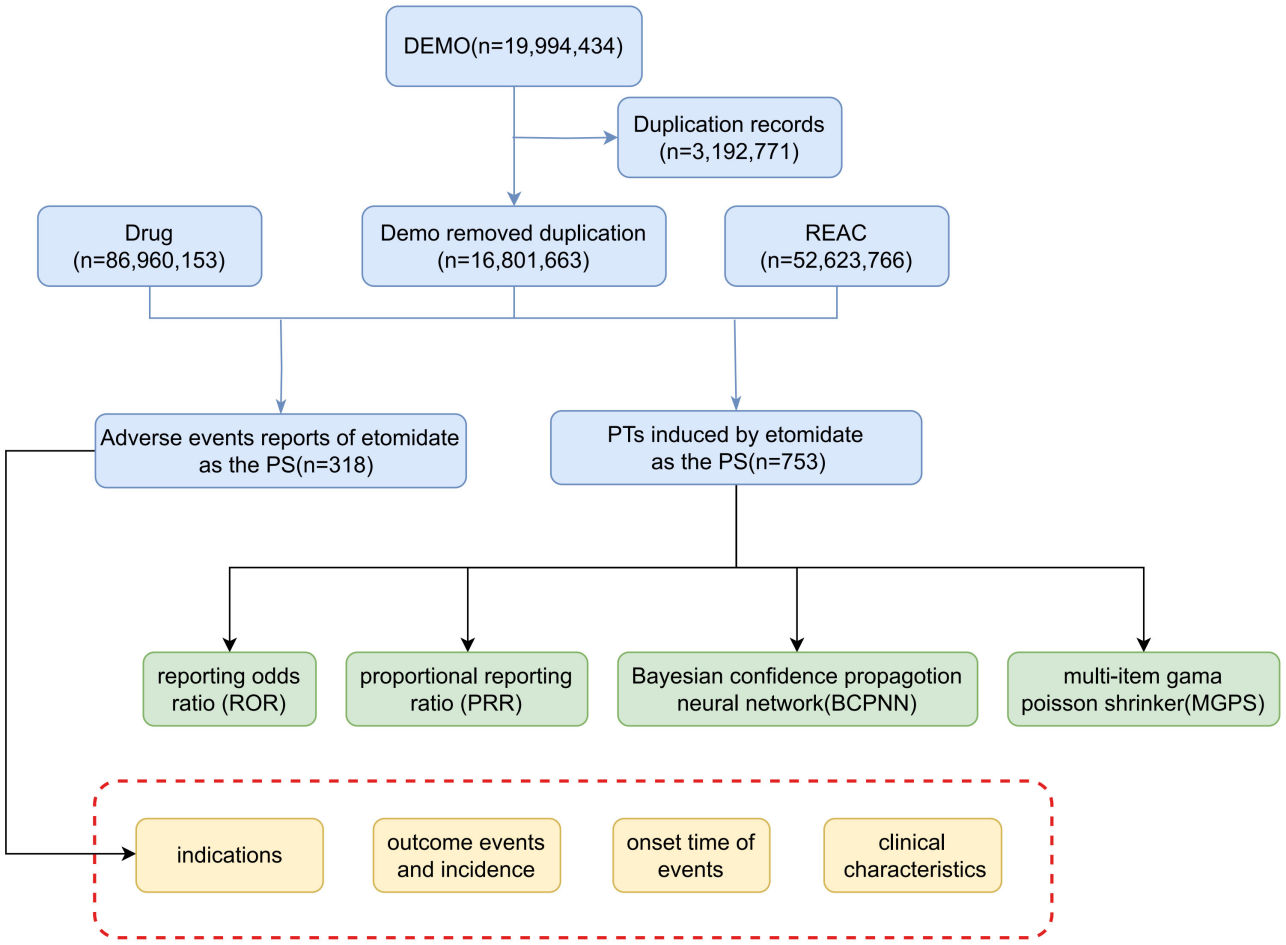
The process flowchart for identifying etomidate-related adverse events from the FAERS database.

In our study, etomidate was identified as the primary suspected drug. Data was retrieved using the faersR package (version 0.0.0.9007), and duplicate records were excluded (Figure 1). The collected AEs were standardized into PTs, which were then further categorized into SOCs according to MedDRA version 26.1. Additional clinical characteristics such as age, time to onset (TTO), and region were also gathered. Serious AEs were defined as those involving hospitalization, life-threatening conditions, disability, and similar outcomes.

### 2.2 Signal detection algorithm and statistics

A disproportionality analysis was conducted to assess the potential link between specific AEs and the suspected drug. Descriptive analysis was utilized to present the baseline characteristics of the AEs associated with the target drug. Frequencies were employed to describe categorical data. For continuous variables with a normal distribution, the mean value was calculated and presented. For those data with a skewed distribution, the median value was used. Using data from the FAERS database, the reporting odds ratio (ROR), proportional reporting ratio (PRR), Bayesian confidence propagation neural network (BCPNN), and empirical Bayesian geometric mean (EBGM) were calculated. The formulas for these four algorithms and the criteria for their selection were provided in the supplementary table (Supplementary Table 1). Additionally, the 95% confidence intervals (CI) for ROR, PRR, BCPNN, and EBGM were also calculated. AEs that met all the criteria were identified as positive signals. In the supplementary table, “a” represents the number of target AEs for the suspected drug, “b” represents the number of other AEs for the suspected drug, “c” represents the number of target AEs for other drugs, and “d” represents the number of other AEs for other drugs (Supplementary Table 2). Data processing and visualization were performed using R (version 4.3.3).

## 3 Results

### 3.1 Baseline profile of etomidate

Etomidate was a hypnotic agent that was frequently used in induction of anesthesia. It could promote the effect of γ-aminobutyric acid by modulating the γ-aminobutyric acid type A receptor. It was firstly introduced into clinical practice as the first non-barbiturate intravenous anesthetic since 1972 (24, 25). In our research, the drug safety profile of etomidate reported in the FAERS database were collected and analyzed. To sum up, a total of 318 adverse events were recorded, most of which were found related to anesthesia (*n* = 43, 13.19%) or induction of anesthesia (*n* = 43, 13.19%). 102 cases (32.08%) were reported from female while 96 (30.19%) were reported from male. 78 cases (24.53%) were recorded from the population under the age of 45 years old, followed by 63 cases (19.81%) from the population aged 45 ~ 65 years old. As for the sources of the AEs, 121 cases (38.05%) were reported by pharmacist, followed by physicians (*n* = 98, 30.82%), other health-professional (*n* = 59, 18.55%) and consumers (*n* = 20, 6.29%). United states has the highest number of reported adverse events in the world (*n* = 160, 82.90%). 108 AEs (33.96%) were reported in intravenous route while most of the rest were recorded from other routes (*n* = 187, 58.81%). 166 AEs (47.70%) of etomidate were reported to develop into other serious outcomes, followed by 59 cases of hospitalization (16.95%) and 54 cases of required intervention to prevent permanent impairment/damage (15.52%). Lastly, the time to onset (TTO) of AEs of etomidate were summarized and it was found that 79 AEs (24.84%) were reported within 30 days since administration (Table 1).

**Table 1:** Features of etomidate-related adverse events reported in the FAERS database from 2004 to 2025.

Variable	*n* (%)
Indications	
Abdominal pain upper	1.00 (0.31)
Acute respiratory failure	1.00 (0.31)
Anesthesia	43.00 (13.19)
Anesthesia procedure	2.00 (0.61)
Asthma	2.00 (0.61)
Atrial fibrillation	1.00 (0.31)
Cardiovascular disorder	1.00 (0.31)
Cardioversion	2.00 (0.61)
Colonoscopy	1.00 (0.31)
Completed suicide	1.00 (0.31)
Coronary artery surgery	1.00 (0.31)
Cushing's syndrome	5.00 (1.53)
Depression	1.00 (0.31)
Drug exposure during pregnancy	4.00 (1.23)
Drug use for unknown indication	3.00 (0.92)
Dyspnoea	1.00 (0.31)
Electroconvulsive therapy	10.00 (3.07)
Endarterectomy	1.00 (0.31)
Endoscopy	1.00 (0.31)
Endotracheal intubation	26.00 (7.98)
Epilepsy	4.00 (1.23)
Explorative laparotomy	1.00 (0.31)
General anesthesia	19.00 (5.83)
Hyperadrenocorticism	1.00 (0.31)
Induction and maintenance of anesthesia	1.00 (0.31)
Induction of anesthesia	43.00 (13.19)
Joint dislocation	1.00 (0.31)
Light anesthesia	2.00 (0.61)
Mental disorder	1.00 (0.31)
Pain	1.00 (0.31)
Poisoning	1.00 (0.31)
Product used for unknown indication	35.00 (10.74)
Resuscitation	1.00 (0.31)
Sedation	27.00 (8.28)
Sedative therapy	23.00 (7.06)
Subarachnoid hemorrhage	1.00 (0.31)
Surgery	2.00 (0.61)
Tachycardia	1.00 (0.31)
Unknown	50.00 (15.34)
Wada test	3.00 (0.92)
Gender	
Female	102.00 (32.08)
Male	96.00 (30.19)
Unknown	120.00 (37.74)
**Mean age (Year)**	47.00 (34.00, 65.00)
Age (Year)	
< 45	78.00 (24.53)
45 ~ 65	63.00 (19.81)
65 ~ 75	34.00 (10.69)
> = 75	14.00 (4.40)
Unknow	129.00 (40.57)
**Weight (kg)**	77.85 (68.53, 92.08)
Reporter	
Pharmacist	121.00 (38.05)
Physician	98.00 (30.82)
Other health-professional	59.00 (18.55)
Consumer	20.00 (6.29)
Unknown	19.00 (5.97)
Registered Nurse	1.00 (0.31)
Reported countries	
United States	160.00 (82.90)
Others	33.00 (17.10)
Route	
Other	187.00 (58.81)
Intravenous	108.00 (33.96)
Intravenous bolus	23.00 (7.23)
Outcomes	
Other serious	166.00 (47.70)
Hospitalization	59.00 (16.95)
Required intervention to prevent permanent impairment/damage	54.00 (15.52)
Life threatening	34.00 (9.77)
Death	29.00 (8.33)
Disability	6.00 (1.72)
**Average TTO (Days)**	0.00 (0.00, 0.00)
TTO group (Days)	
< 30	79.00 (24.84)
31 ~ 60	1.00 (0.31)
61 ~ 90	1.00 (0.31)
91 ~ 180	0.00 (0.00)
181 ~ 360	0.00 (0.00)
> = 360	0.00 (0.00)
Unknow	14.00 (4.40)

TTO, time to onset.

### 3.2 The signal detection of AEs about etomidate

#### 3.2.1 SOCs associated with etomidate

**Figure 2:**
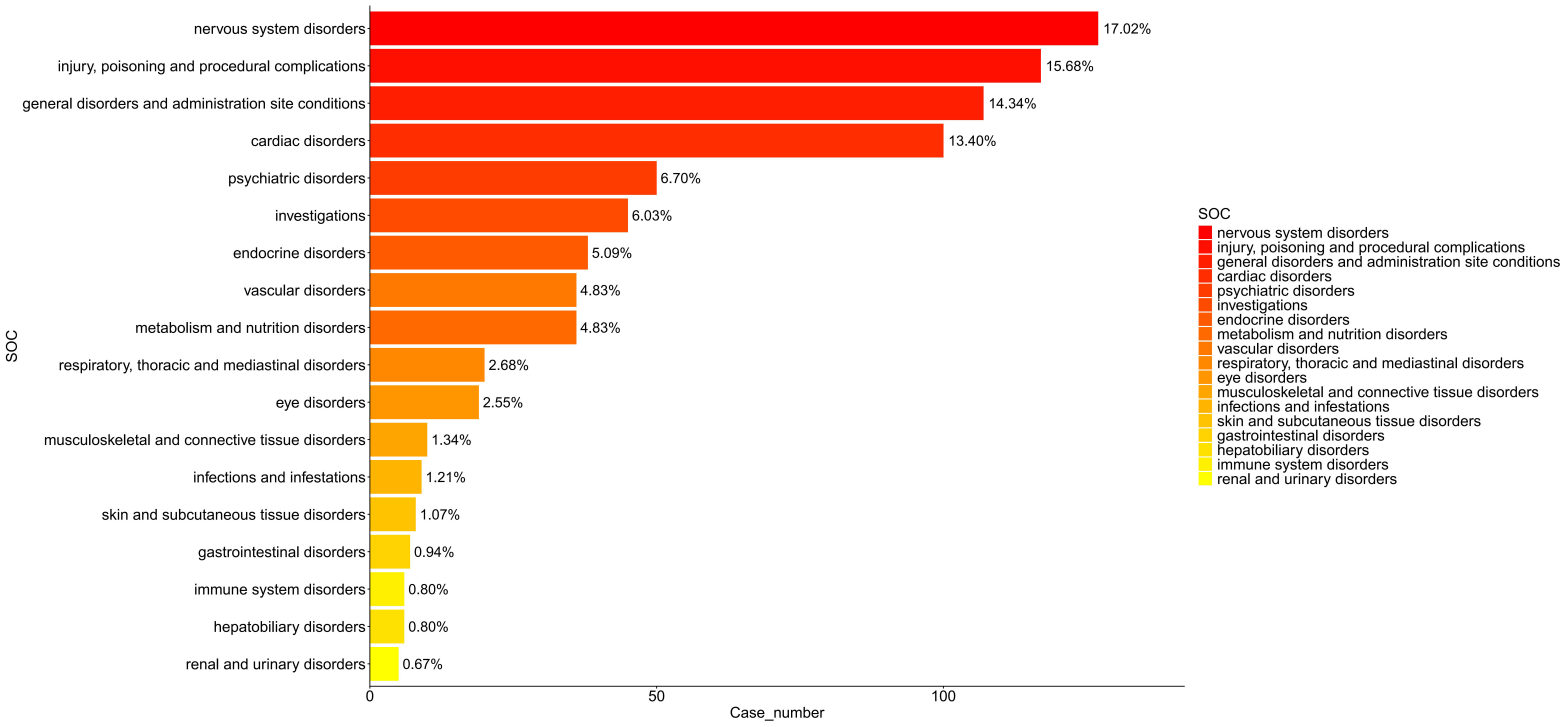
The bar chart summarizing the 18 SOCs associated with etomidate. The proportion of each type of SOC was calculated and presented as percentage values. (SOC, system organ class).

Based on the analysis, the AEs were categorized into 18 SOCs related to the use of etomidate. The SOCs with the highest number of reports included nervous system disorders (*n* = 127, 17.02%), injury, poisoning, and procedural complications (*n* = 117, 15.68%), general disorders and administration site conditions (*n* = 107, 14.34%), cardiac disorders (n=100, 13.40%) and psychiatric disorders (*n* = 50, 6.70%) (Figure 2). Among these, the SOCs that met the four algorithm criteria were cardiac disorders (*n* = 100, 13.40%) and endocrine disorders (*n* = 38, 5.09%). Compared with the existing drug inserts, cardiac disorders (*n* = 100, 13.40%) was shown to be associated with etomidate in our study, which underscores the robustness of our research. Notablely, endocrine disorders (*n* = 38, 5.09%) was uncovered as a positive signal associated with the use of etomidate (Supplementary Table 3). Even though adrenal suppression is a well known AE of etomidate, other potential AEs of endocrine system like thyrotoxic crisis were reported in our post-market surveillance. Continuous monitoring should be encouraged and it could help in drug improvements, which could further reduce anesthesia-related risks.

#### 3.2.2 PTs associated with etomidate

**Figure 3:**
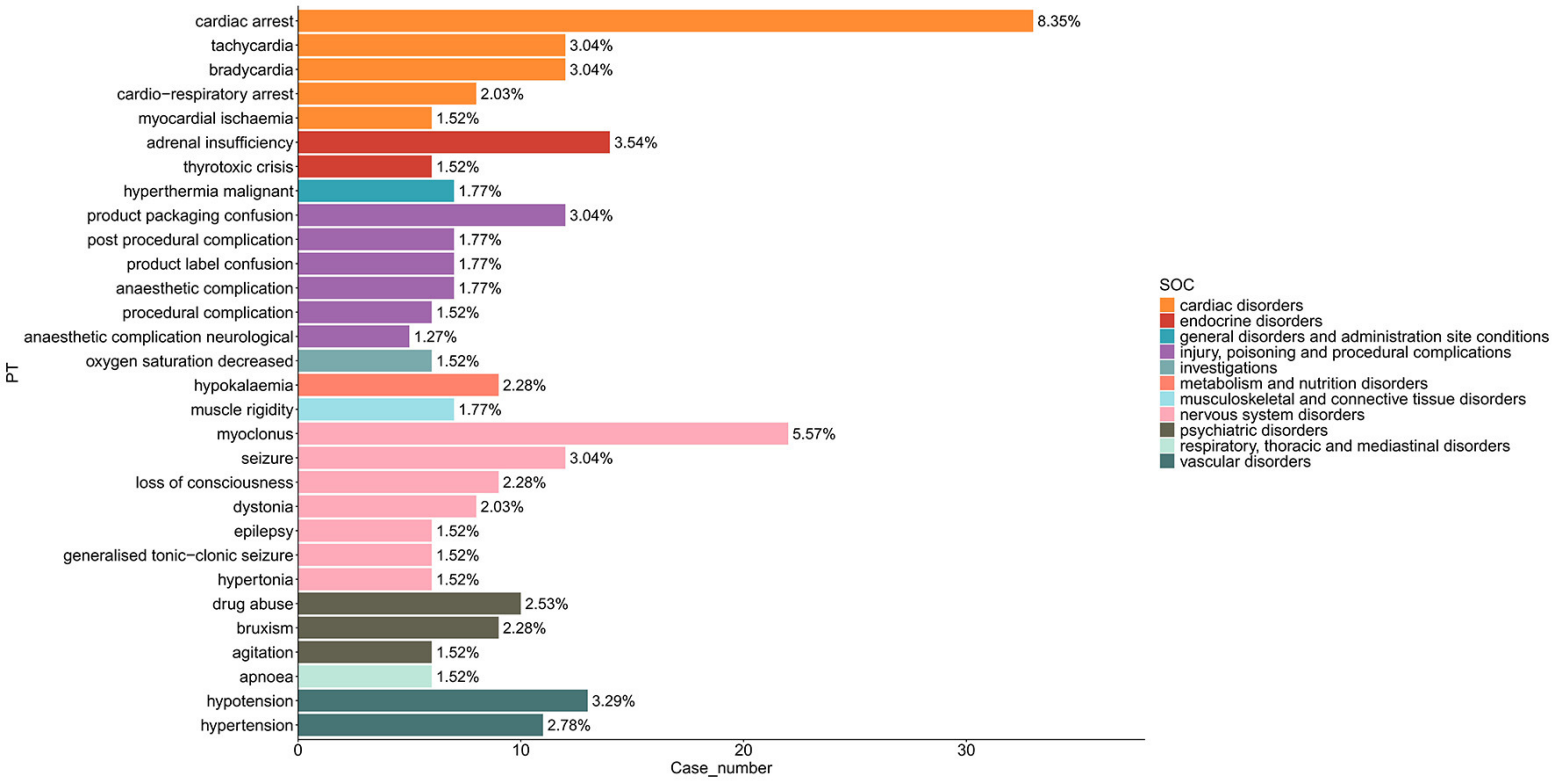
Bar plot showing the top 30 adverse events for etomidate in the FAERS database, organized by PTs for case number. Colors indicated the SOC associated with each PT. The proportions of each item were presented as percentage values. (SOC, system organ class; PT, preferred term).

Based on the results of the four algorithms, 59 PTs were identified to be significantly associated with etomidate. Top 30 were demonstrated and classified into 11 different SOCs in the barplot (Figure 3). According to the case number reported in the database, it was found that cardiac arrest (*n* = 33, 8.35%) had the higher proportion of records than others, followed by myoclonus (*n* = 22, 5.57%), adrenal insufficiency (*n* = 14, 3.54%), hypotension (*n* = 13, 3.29%), and product packaging confusion (*n* = 12, 3.04%), which was consistent with the drug's package insert and published research reports.

To our surprise, some AEs undocumented on drug inserts were identified as positive signals in our study. As shown in the supplementary table (Supplementary Table 4), cardiac arrest (*n* = 33) seizure (*n* = 12), drug abuse (*n* = 10), hypokalaemia (*n* = 9), bruxism (*n* = 9), hyperthermia malignant (*n* = 7), epilepsy (*n* = 6), agitation (*n* = 6), thyrotoxic crisis (*n* = 6), and anesthetic complication neurological (*n* = 5), etc. were found to be associated with etomidate. Even though the incidence of these AEs were relatively low, they deserves attention in clinical practice.

## 4 Discussion

Based on our analysis of the FAERS database, this study provided a comprehensive post-marketing safety profile of etomidate. The main findings revealed that etomidate associated AEs most frequently involve the nervous system, injuries and procedural complications, general disorders, and cardiac disorders. Notably, cardiac disorders and endocrine disorders met all four signal detection algorithms, with the latter representing a novel positive signal beyond the well-documented adrenal suppression (26). Additionally, several significant PTs including cardiac arrest, myoclonus, and adrenal insufficiency were consistent with known safety information. To our surprise, some novel positive signals such as seizure, drug abuse, hypokalaemia, bruxism, malignant hyperthermia, epilepsy, agitation, thyrotoxic crisis, and anesthetic complication neurological were identified. These findings underscore the importance of continuous pharmacovigilance to enhance the understanding of etomidate's risk profile in clinical practice.

Consistent with the pharmacovigilance study conducted in Korea (27), cardiac disorders were also identified as a positive signal associated with etomidate in our analysis, which proved the reliability of our analysis. Although both tachycardia and bradycardia are known as potential AEs with etomidate administration, it is noteworthy that our analysis revealed cardiac arrest to be a significant signal. For vulnerable patients, etomidate should be administered with extreme caution for anesthesia induction to prevent cardiac arrest, particularly in those with adrenal insufficiency (28). Drug induced hypotension and circulatory collapse may contribute to cardiac arrest, but the exact mechanisms remain to be further investigated. Seizure was found to be a positive signal in the study and it was consistent with published case reports (29, 30). It was demonstrated in some studies that etomidate could activate high-frequency oscillations and spike activity in epilepsy patients, which are consistent with the electrophysiological characteristics of the seizure onset zone (31, 32). The results of our analysis confirm that seizures induced by etomidate represent an adverse event that warrants attention. From a clinical observational standpoint, it should be emphasized that the excitatory effects of etomidate may occasionally be misinterpreted as epileptic seizures. This phenomenon can be counteracted by concomitant administration of neuromuscular blocking drugs, which necessitates careful differentiation and close monitoring by clinicians. When etomidate is used for the localization of epileptic foci in the brain, it is essential to be vigilant about its potential to induce and exacerbate seizures. Historically, etomidate was administered as a continuous infusion to control refractory status epilepticus. Seizures were terminated by suppressing cortical activity under close EEG monitoring, but this was a therapeutic—not a preventive—measure and carried severe adverse effects such as adrenal suppression, so it is now rarely used (33). Later it was proposed that etomidate induced myoclonus may represent a form of seizure and use of butorphanol prior to etomidate administration was effective in seizure prevention (34). Since 2011, following the classification of propofol as a controlled anesthetic in South Korea and other regions, the circulation and abuse of etomidate as a substitute in the illicit market have increased dramatically (35). In June 2020, the Ministry of Food and Drug Safety of South Korea included etomidate in the category of “drugs of concern for potential abuse or misuse” under regulatory management. Several studies have found that etomidate is being added to conventional cigarette tobacco (commonly referred to as “cigarette powder”) or mixed into e-liquid, and sold through channels such as the internet, retail stores, or entertainment venues (36–39). An animal study directly demonstrated the rewarding effect and reinforcing effect of etomidate through conditioned place preference (CPP) and self-administration experiments, suggesting its potential for drug addiction (40). It was revealed in zebrafish that etomidate exposure will lead to its accumulation in the brain, elevate GABA levels, and promote oxidative stress. These effects subsequently cause mitochondrial swelling, rupture, and damage to myelinated nerve fibers, ultimately resulting in brain injury manifested as reduced anxiety, memory impairment, and heightened aggression (41). A study based on mouse model confirmed that long-term etomidate intake significantly reduced serotonin and GABA levels in the brain. High doses of etomidate increased neuronal apoptosis and potentially induced drug resistance and dependence. Additionally, etomidate exposure caused intestinal damage and gut microbiota disruption, while also triggering abnormal glycerophospholipid metabolism in both the colon and the brain. These may lead to the accumulation of lipotoxic metabolites and impair the central nervous system (42). Analysis of urinary steroid profiles in etomidate abusers revealed suppressed 11β-hydroxylase activity, indicating that chronic abuse may cause acquired 11β-hydroxylase deficiency. This significantly impairs adrenal cortical function and increases the risk of hypokalemia and hyperandrogenism (43). Bruxism is defined as a repetitive jaw-muscle activity characterized by clenching or grinding of the teeth and/or by bracing or thrusting of the mandible (44). It is reported that the level of gamma-aminobutyric acid (GABA) is associated with bruxism. Substances with affinity for GABA or structural analogs, such as clonazepam, tiagabine, and gabapentin, have been shown to reduce the occurrence of bruxism (45). However, studies have also shown that GABA receptors are present in the mesencephalic trigeminal nucleus (MTN). During sleep, the inhibitory neurotransmitter GABA released from the ventrolateral preoptic area of the hypothalamus can activate the MTN to release glutamate, which subsequently activates the ascending reticular activating system (ARAS). This process ultimately induces rhythmic masticatory muscle activity (RMMA)—the physiological basis of sleep bruxism (46). Therefore, we hypothesize that etomidate, through its GABA-like effects, may mediate the occurrence of bruxism via the MTN–ARAS–sleep micro-arousal neural circuit. To date, no studies have confirmed that the use of etomidate could induce bruxism and it warrants further validation. As for malignant hyperthermia, etomidate itself is not a potent trigger. In non-susceptible individuals or patients with specific diseases, it generally does not induce fever and is considered a safe induction agent. However, animal studies have confirmed that in malignant hyperthermia-susceptible pigs, although etomidate infusion did not directly cause malignant hyperthermia, it significantly increased body temperature and elevated plasma lactate levels (47). Temperature monitoring is of great significance during the administration of etomidate. Clinical observations have revealed that the incidence of postoperative agitation following general anesthesia induction with etomidate was 8.75%, whereas no such cases were observed with propofol (48). It was demonstrated that etomidate-induced anesthesia was a significant risk factor for the development of “emergence delirium” postoperatively (OR = 2.7, *p* < 0.001), which primarily manifests as agitation and confusion (49). Furthermore, multiple studies on electroconvulsive therapy (ECT) have also indicated an association between the use of etomidate and a higher incidence of postoperative agitation, which required the administration of benzodiazepines and clonidine to control post-ECT agitation (50–52). It was indicated that etomidate may induce central anticholinergic syndrome (CAS), manifesting as agitation, hallucinations, disorientation, among other symptoms (53). Thyrotoxic crisis is a life-threatening endocrine emergency, the occurrence of which may be related to a sharp increase in circulating thyroid hormone levels. It mostly occurs in patients with severe or long-standing hyperthyroidism. They have not received treatment or have been inadequately treated. Our study indicated a potential association between etomidate and thyroid storm, which underscored the importance of perioperative thyroid function assessment when using etomidate. However, there is currently no direct evidence to confirm such a relationship. Therefore, further validation through large-scale cohort study is warranted. Neurological complication was found to be associated with etomidate. Apart from the known myoclonus, previous studies have demonstrated that etomidate induced neuronal apoptosis and alters the expression of genes associated with learning and memory, including Arc, c-fos, and Egr1, thereby contributing to cognitive dysfunction (54, 55). Besides, it was reported that chronic exposure to etomidate may induce motor impairments, neurotransmitter dysregulation, oxidative stress, and neuroinflammatory responses in zebrafish models, thereby highlighting its potential role as a developmental neurotoxicant (56). These AEs were firstly uncovered associated with the administration of etomidate, which suggest that we should continuously monitor newly identified adverse events to better regulate the drug's use.

The study has several limitations that warrant consideration. First, the analysis relied on retrospective pharmacovigilance data from the FAERS database, which is inherently subject to underreporting, reporting bias, and incomplete clinical details, thus limiting causal inference. For instance, the FAERS database does not provide further sub classification of the patient population receiving etomidate; therefore, data regarding the use of this agent in critically ill patients remain unavailable. Second, variability in patient characteristics, comorbidities, and concomitant medications could not be fully adjusted for, potentially confounding the observed associations. Third, although disproportionality analyses improve signal detection, they do not provide estimates of true incidence or risk magnitude. Furthermore, the absence of experimental or clinical validation constrains mechanistic interpretation. Future investigations should incorporate prospective clinical studies, mechanistic animal models, and large multicenter real-world cohorts to validate these signals, clarify underlying pathways, and refine strategies for safer clinical application of etomidate.

The results of this study reinforce the need for risk management of etomidate in the field of anesthesia, particularly in high-risk populations. Future research should further explore the mechanisms of these adverse events and work toward drug improvements or the development of new medications to reduce anesthesia-related risks. Although etomidate has significant value in clinical practice, its safety must be closely monitored in actual operations to ensure safety.

## 5 Conclusions

This study provided a comprehensive post-marketing safety assessment of etomidate using real-world data from the FAERS database. In addition to confirming established adverse events, such as adrenal suppression, myoclonus, and cardiovascular complications, we identified several novel safety signals, including seizures, endocrine disorders, bruxism, and thyrotoxic crisis. These findings underscore the multifaceted risk profile of etomidate and highlight the necessity of continuous pharmacovigilance, particularly in vulnerable populations. While etomidate remains a clinically valuable anesthetic due to its favorable hemodynamic properties, its use should be guided by careful risk–benefit evaluation and vigilant monitoring. Future prospective studies, mechanistic investigations, and multicenter cohort analyses are warranted to validate these signals and to inform evidence-based strategies for optimizing the safe clinical application of etomidate.

## Data Availability

The original contributions presented in the study are included in the article/Supplementary material, further inquiries can be directed to the corresponding authors.

## References

[1] Kay B. A dose-response relationship for etomidate, with some observations on cumulation. Br J Anaesth. (1976) 48:213–6. doi: 10.1093/bja/48.3.213 1259887

[2] Criado A Maseda J Navarro E Escarpa A Avello F. Induction of anaesthesia with etomidate: haemodynamic study of 36 patients. Br J Anaesth. (1980) 52:803–6. doi: 10.1093/bja/52.8.803 7426258

[3] Egan ED Johnson KB. The influence of hemorrhagic shock on the disposition and effects of intravenous anesthetics: a narrative review. Anesth Analg. (2020) 130:1320–30. doi: 10.1213/ANE.0000000000004654 32149755

[4] Uhm J Hong S Han E. The need to monitor emerging issues in etomidate usage: the misuse or abuse potential. Forensic Sci Med Pathol. (2024) 20:249–60. doi: 10.1007/s12024-023-00596-4 36853502

[5] Kim MG Park SW Kim JH Lee J Kae SH Jang HJ . Etomidate versus propofol sedation for complex upper endoscopic procedures: a prospective double-blinded randomized controlled trial. Gastrointest Endosc. (2017) 86:452–61. doi: 10.1016/j.gie.2017.02.033 28284883

[6] Lee JM Min G Keum B Lee JM Kim SH Choi HS . Using etomidate and midazolam for screening colonoscopies results in more stable hemodynamic responses in patients of all ages. Gut Liver. (2019) 13:649–57. doi: 10.5009/gnl18514 30970436 PMC6860030

[7] Fuchs-Buder T Sparr HJ Ziegenfuss T. Thiopental or etomidate for rapid sequence induction with rocuronium. Br J Anaesth. (1998) 80:504–6. doi: 10.1093/bja/80.4.504 9640158

[8] Morris C McAllister C. Etomidate for emergency anaesthesia; mad, bad and dangerous to know? Anaesthesia. (2005) 60:737–40. doi: 10.1111/j.1365-2044.2005.04325.x 16029220

[9] Ledingham IM Watt I. Influence of sedation on mortality in critically ill multiple trauma patients. Lancet. (1983) 1:1270. doi: 10.1016/S0140-6736(83)92712-5 6134053

[10] Sunshine JE Deem S Weiss NS Yanez ND Daniel S Keech K . Etomidate, adrenal function, and mortality in critically ill patients. Respir Care. (2013) 58:639–46. doi: 10.4187/respcare.01956 22906838 PMC4126750

[11] Office SP. A Drug-Crime White Paper 2017 Chapter 1. (2021). Available online at: https://www.spo.go.kr/site/spo/ex/board/ List.do?cbIdx=1204 (accessed August 19, 2021).

[12] Safety MoFaD. Report On Regulatory Impact Analysis (Regulation On The Designation Of Drugs That May Cause Concerns Of Misuse Or Abuse). (2021). Available online at: https:// www.mfds.go.kr/brd/m_209/view.do?seq=43304&srchFr=& srchTo=&srchWord=&srchTp=&itm_seq_1=0&itm_seq_2= 0&multi_itm_seq=0&company_cd=&company_nm=&page=2 (accessed August 19, 2021).

[13] Xie W Zhou L Liu J Li Z Li Z Gao W . How to trace etomidate in illegal E-cigarettes from authentic human hair: identification, quantification and multiple-factor analysis. Forensic Toxicol. (2024) 107–108. doi: 10.1007/s11419-024-00698-w 39122972

[14] Online D. Your Guide to Quality Drug Data, Etomidate. (2021). Available online at: https://go.drugbank.com/drugs/DB00292 (accessed August 19 2021).

[15] Crozier TA Beck D Schlaeger M Wuttke W Kettler D. Endocrinological changes following etomidate, midazolam, or methohexital for minor surgery. Anesthesiology. (1987) 66:628–35. doi: 10.1097/00000542-198705000-00006 3034107

[16] Absalom A Pledger D Kong A. Adrenocortical function in critically ill patients 24 h after a single dose of etomidate. Anaesthesia. (1999) 54:861–7. doi: 10.1046/j.1365-2044.1999.01003.x 10460557

[17] Wunsch H Bosch NA Law AC Vail EA Hua M Shen BH . Evaluation of etomidate use and association with mortality compared with ketamine among critically Ill patients. Am J Respir Crit Care Med. (2024) 116–119. doi: 10.1164/rccm.202404-0813OC 39173173

[18] Komatsu R You J Mascha EJ Sessler DI Kasuya Y Turan A. Anesthetic induction with etomidate, rather than propofol, is associated with increased 30-day mortality and cardiovascular morbidity after noncardiac surgery. Anesth Analg. (2013) 117:1329–37. doi: 10.1213/ANE.0b013e318299a516 24257383

[19] Watt I Ledingham IM. Mortality amongst multiple trauma patients admitted to an intensive therapy unit. Anaesthesia. (1984) 39:973–81. doi: 10.1111/j.1365-2044.1984.tb08885.x 6496912

[20] Morris R Ali R Cheng F. Drug repurposing using FDA adverse event reporting system (FAERS) database. Curr Drug Targets. (2024) 25:454–64. doi: 10.2174/0113894501290296240327081624 38566381

[21] Sun S Zhang Y Wu H Peng W. Analysis of lumateperone data for patients with schizophrenia using related adverse events from the FDA adverse reporting system. Expert Opin Drug Saf. (2024). doi: 10.1080/14740338.2024.2392869 39193998

[22] Zhao K Zhao Y Xiao S Tu C. Assessing the real-world safety of tralokinumab for atopic dermatitis: insights from a comprehensive analysis of FAERS data. Front Pharmacol. (2024) 15:1458438. doi: 10.3389/fphar.2024.1458438 39193341 PMC11347326

[23] Zou D Hu Q Liu Y Yu L. Post-marketing pharmacovigilance study of inclisiran: mining and analyzing adverse event data from the FDA adverse event reporting system database. Int J Clin Pharm. (2024). doi: 10.1007/s11096-024-01784-0 39192158

[24] Doenicke A. Etomidate, a new intravenous hypnotic. Acta Anaesthesiol Belg. (1974) 25:307–15. 4143092

[25] Janssen PA Niemegeers CJ Marsboom RP. Etomidate, a potent non-barbiturate hypnotic. Intravenous etomidate in mice, rats, guinea-pigs, rabbits and dogs. Arch Int Pharmacodyn Ther. (1975) 214:92–132. 1156027

[26] Greer A Hewitt M Khazaneh PT Ergan B Burry L Semler MW . Ketamine versus etomidate for rapid sequence intubation: a systematic review and meta-analysis of randomized trials. Crit Care Med. (2024) 53:e374–83. doi: 10.1097/CCM.0000000000006515 39570063

[27] Choi YJ Yang SW Kwack WG Lee JK Lee TH Jang JY . Comparative safety profiles of sedatives commonly used in clinical practice: a 10-year nationwide pharmacovigilance study in Korea. Pharmaceuticals. (2021) 14. doi: 10.3390/ph14080783 34451882 PMC8399659

[28] Mathur R Boyadzhyan A Mora A Afaq S. Acute adrenal crisis following etomidate administration in a patient with preexisting adrenal insufficiency. Cureus. (2025) 17:e88003. doi: 10.7759/cureus.88003 40821333 PMC12352522

[29] Rainess RA Patel V Stander E. Etomidate induced seizure: adverse drug event case report. J Pharm Pract. (2020) 35:126-−8. doi: 10.1177/0897190020958243 32924746

[30] Sen H Algul A Senol MG Ates A Kilic E Ozkan S . Epileptic seizure during anaesthesia induction with etomidate. Middle East J Anaesthesiol. (2010) 20:723–5. 20803863

[31] Vega-Zelaya L Pastor J Tormo I de Sola RG Ortega GJ. Assessing the equivalence between etomidate and seizure network dynamics in temporal lobe epilepsy. Clin Neurophysiol. (2015) 127:169–78. doi: 10.1016/j.clinph.2015.05.008 26070516

[32] Rampp S Schmitt HJ Heers M Schönherr M Schmitt FC Hopfengärtner R . Etomidate activates epileptic high frequency oscillations. Clin Neurophysiol. (2013) 125:223–30. doi: 10.1016/j.clinph.2013.07.006 23911722

[33] Yeoman P Hutchinson A Byrne A Smith J Durham S. Etomidate infusions for the control of refractory status epilepticus. Intensive Care Med. (1989) 15:255–9. doi: 10.1007/BF00271062 2745868

[34] He L Ding Y Chen H Qian Y Li Z. Butorphanol pre-treatment prevents myoclonus induced by etomidate: a randomised, double-blind, controlled clinical trial. Swiss Med Wkly. (2014) 144:w14042. doi: 10.4414/smw.2014.14042 25317545

[35] Zheng G Chen Y Wu G Song T Zou X Nie Q . Review of the hazards and contraindications of etomidate. Int J Toxicol. (2024) 44:245–53. doi: 10.1177/10915818241297073 39501904

[36] Liying Z Junbo Z Wanting X Ping X Yan S Hejian W . Detection of “smoke powder” etomidate and its metabolite etomidate acid in blood and urine by UHPLC-MS-MS: application in authentic cases. J Anal Toxicol. (2024) 48:701–9. doi: 10.1093/jat/bkae080 39340313

[37] Lin M Zhang Z He Q Hao H Xiang P Zhao J. Rapid determination of etomidate and its structural analogues in e-liquid by probe electrospray ionization quadrupole time-of-flight mass spectrometry. J Pharm Biomed Anal. (2025) 256:116677. doi: 10.1016/j.jpba.2025.116677 39823969

[38] Zhang X Li Y Liu J Pan Z Chen Y Wang M . Identification of two novel imidazole-derived GABA agonists butomidate and tf-etomidate in e-cigarette liquids. Forensic Toxicol. (2025) 44:47–60. doi: 10.1007/s11419-025-00732-5 40643820

[39] Mo Y Zhang X Zou K Xing W Hou X Zeng Y . Au ordered array substrate for rapid detection and precise identification of etomidate in e-liquid through surface-enhanced Raman spectroscopy. Nanomaterials. (2024) 14:1958. doi: 10.3390/nano14231958 39683346 PMC11643652

[40] Kuai L Li X Xu D Zeng L Xu P Di B . Behavioral studies of the abuse potential and anesthetic and sedative effects of etomidate in male rodents. Psychopharmacology. (2024) 242:641–9. doi: 10.1007/s00213-024-06715-5 39527141

[41] Li X Lin X Zhang Z Zhuang Z Li Y Luo Y . Neurotoxicity and aggressive behavior induced by anesthetic etomidate exposure in zebrafish: Insights from multi-omics and machine learning. Aquat Toxicol. (2025) 282:107321. doi: 10.1016/j.aquatox.2025.107321 40068374

[42] Ding S Li K Han X Lin W Qin Y Cao R . Long-term use of etomidate disrupts the intestinal homeostasis and nervous system in mice. Toxicology. (2024) 504:153802. doi: 10.1016/j.tox.2024.153802 38604439

[43] Cheung YT Lau CY Tseung JS Yu KY Cheung HN Shek CC . Acquired 11β-hydroxylase deficiency in etomidate and (Iso)propoxate abusers: a nascent endocrine condition. Steroids. (2025) 220:109639. doi: 10.1016/j.steroids.2025.109639 40451601

[44] Lobbezoo F Ahlberg J Glaros AG Kato T Koyano K Lavigne GJ . Bruxism defined and graded: an international consensus. J Oral Rehabil. (2012) 40:2–4. doi: 10.1111/joor.12011 23121262

[45] Lavigne GJ Khoury S Abe S Yamaguchi T Raphael K. Bruxism physiology and pathology: an overview for clinicians. J Oral Rehabil. (2008) 35:476–94. doi: 10.1111/j.1365-2842.2008.01881.x 18557915

[46] Giovanni A Giorgia A. The neurophysiological basis of bruxism. Heliyon. (2021) 7:e07477. doi: 10.1016/j.heliyon.2021.e07477 34286138 PMC8273205

[47] Suresh MS Nelson TE. Malignant hyperthermia: is etomidate safe? Anesth Analg. (1985) 64:420–4. doi: 10.1213/00000539-198504000-00009 3985391

[48] Weng D Huang M Jiang R Zhan R Yang C. Clinical study of etomidate emulsion combined with remifentanil in general anesthesia. Drug Des Devel Ther. (2013) 7:771–6. doi: 10.2147/DDDT.S45979 23990706 PMC3753064

[49] Radtke FM Franck M Hagemann L Seeling M Wernecke KD Spies CD. Risk factors for inadequate emergence after anesthesia: emergence delirium and hypoactive emergence. Minerva Anestesiol. (2010) 76:394–403. 20473252

[50] Freeman SA. Post-electroconvulsive therapy agitation with etomidate. J ECT. (2009) 25:133–4. doi: 10.1097/YCT.0b013e3181872728 19494736

[51] Li KJ Slama NE Hirschtritt ME Anshu P Iturralde E. Electroconvulsive therapy anesthetic choice and clinical outcomes. J ECT. (2022) 39:102–5. doi: 10.1097/YCT.0000000000000895 36729716 PMC10578333

[52] Millischer V Pramhas S Wiedermann I Eder V Kress HG Michalek-Sauberer A . Comparison of etomidate and methohexital as anesthetic agents for continuation and maintenance electroconvulsive therapy: a retrospective analysis of seizure quality and safety. J Affect Disord. (2023) 330:33–9. doi: 10.1016/j.jad.2023.02.085 36863475

[53] Schneck HJ Rupreht J. Central anticholinergic syndrome (CAS) in anesthesia and intensive care. Acta Anaesthesiol Belg. (1989) 40:219–28. 2683549

[54] Chen H-T Zhou J Fan YL Lei CL Li BJ Fan LX . Anesthetic agent etiomidate induces apoptosis in N2a brain tumor cell line. Mol Med Rep. (2018) 18:3137–42. doi: 10.3892/mmr.2018.9298 30066945 PMC6102749

[55] Li X Lu F Li W Xu J Sun XJ Qin LZ . Underlying mechanisms of memory deficits induced by etomidate anesthesia in aged rat model: critical role of immediate early genes. Chin Med J. (2016) 129:48–53. doi: 10.4103/0366-6999.172570 26712432 PMC4797542

[56] Zhuang Z Li X Luo Y Li Y Ahmed Isse S Zhang Z . Developmental neurotoxicity of anesthetic etomidate in zebrafish larvae: alterations in motor function, neurotransmitter signaling, and lipid metabolism. J Hazard Mater. (2025) 494:138598. doi: 10.1016/j.jhazmat.2025.138598 40373404

