# Supplementary material for: Beyond adrenal suppression: a comprehensive post-marketing safety profile of etomidate from real world data
Source: Front Med (Lausanne). 2026 Jul 7;13:1780171. doi: 10.3389/fmed.2026.1780171 (PMC13385346; doi:10.3389/fmed.2026.1780171)
Supplement: Supplementary file 2 [file Table_2.docx]

**Supplementary Table 2. Fourfold table of disproportionality method.**

|  | Drug-related ADEs | Non-drug-related ADEs | Total |
| --- | --- | --- | --- |
| Drug | a | b | a + b |
| Non-drug | c | d | c + d |
| Total | a + c | b + d | N = a + b + c + d |
